# Differential effects of temperature and mTOR and Wnt-planar cell polarity pathways on syndecan-4 and CD44 expression in growth-selected turkey satellite cell populations

**DOI:** 10.1371/journal.pone.0281350

**Published:** 2023-02-03

**Authors:** Jiahui Xu, Gale M. Strasburg, Kent M. Reed, Nora M. Bello, Sandra G. Velleman

**Affiliations:** 1 Department of Animal Sciences, The Ohio State University, Wooster, OH, United States of America; 2 Department of Food Science and Human Nutrition, Michigan State University, East Lansing, MI, United States of America; 3 Department of Veterinary and Biomedical Sciences, University of Minnesota, St. Paul, MN, United States of America; 4 Department of Animal Sciences, The Ohio State University, Columbus, OH, United States of America; UCL: University College London, UNITED KINGDOM

## Abstract

Satellite cells (SCs) comprise a heterogeneous population of muscle stem cells. Thermal stress during the first week after hatch alters proliferation, myogenesis, and adipogenesis of SCs of turkey *pectoralis major* (*p*. *major*) muscle via mechanistic target of rapamycin (mTOR) and wingless-type mouse mammary tumor virus integration site family/planar cell polarity (Wnt/PCP) pathways. Pivotal genes in mTOR and Wnt/PCP pathways are *mTOR* and *frizzled-7* (*Fzd7*), respectively. The objective of this study was to determine the differential effects of thermal stress on SDC4 and CD44 expression in turkey *p*. *major* muscle SCs and how the expression of SDC4 and CD44 is modulated by the mTOR and Wnt/PCP pathways. Satellite cells were isolated from the *p*. *major* muscle of 1-week-old faster-growing modern-commercial (NC) turkeys and slower-growing historic Randombred Control Line 2 (RBC2) turkeys, and were challenged with hot (43°C) and cold (33°C) thermal stress for 72 h of proliferation followed by 48 h of differentiation. The NC line SCs were found to contain a lower proportion of SDC4 positive and CD44 negative (SDC4^+^CD44^−^) cells and a greater proportion of SDC4 negative and CD44 positive (SDC4^−^CD44^+^) cells compared to the RBC2 line at the control temperature (38°C) at both 72 h of proliferation and 48 h of differentiation. In general, at 72 h of proliferation, the proportion of SDC4^+^CD44^−^ cells decreased with heat stress (43°C) and increased with cold stress (33°C) relative to the control temperature (38°C) in both lines, whereas the proportion of SDC4^−^CD44^+^ cells increased with heat stress and decreased with cold stress. In general, the expression of SDC4 and CD44 in the NC SCs showed greater response to both hot and cold thermal stress compared to the RBC2 cells. Knockdown of *mTOR* or *Fzd7* expression increased the proportion of SDC4^+^CD44^−^ cells while the proportion of SDC4^−^CD44^+^ cells decreased during differentiation with line differences being specific to treatment temperatures. Thus, differential composition of *p*. *major* muscle SCs in growth-selected commercial turkey may be resulted, in part, from the alteration in SDC4 and CD44 expression. Results indicate differential temperature sensitivity and mTOR and Wnt/PCP pathway responses of growth-selected SC populations and this may have long-lasting effect on muscle development and growth.

## 1. Introduction

As homeotherms, birds maintain body temperature within a limited range [1]. Newly hatched chickens and turkeys have an immature thermal regulatory system [2, 3], and thus, are susceptible to hot or cold temperature extremes. Thermal stress after hatch changes muscle morphology by altering myofiber diameter, connective tissue spacing between myofibers [4, 5], intramuscular fat deposition [4, 6], and capillary density [7], particularly in the pectoralis major (*p*. *major*; breast) muscle of poultry. With intensive genetic selection, modern faster-growing birds have increased body weight and breast muscle yield at harvest compared to historic slower-growing breeds [8–10]. However, faster-growing birds generate more heat due to increased metabolic rate [11], and lowered capacity for heat dissipation caused by reduced capillary density in the *p*. *major* muscle [7, 12]. Thus, under heat stress, faster-growing poultry have a decreased ability to maintain body temperature [13], resulting in increased myofiber degeneration [7] and increased fat deposition [6] in the *p*. *major* muscle compared to slower-growing lines.

Myofiber formation is complete by the time of hatch [14], and post-hatch muscle growth occurs through the hypertrophy of existing myofibers. Satellite cells (SCs) are adult myoblasts that drive myofiber hypertrophy through proliferation, differentiation, and donation of their nuclei to existing myofibers [15, 16]. During the first week after hatch in poultry, SCs have the greatest mitotic activity [17, 18] and their proliferation and differentiation are highly responsive to thermal stress [19]. As multipotential stem cells [20, 21], SCs can convert to an adipocyte-like lineage in response to external heat stress [22–24]. Thermal stress immediately after hatch has long-lasting effects on *p*. *major* muscle development, growth, and fat deposition, and this has been associated with altered function and fate of SCs [4, 5, 25].

Satellite cells comprise a heterogenous population of muscle stem cells [26–30] with unique SC populations observed in different types of adult myofibers [31]. Khodabukus and Baar [32] reported that SCs from slow and fast myofibers express slow and fast contractile proteins and can inherit metabolic properties of their native myofibers. Satellite cell populations can also vary among myofibers in the same muscle. For example, SCs isolated from the *p*. *major* muscle of one turkey have different rates of proliferation and differentiation [33] and different sensitivities to growth factors [34, 35]. In the rat flexor digitorum brevis muscle, SCs with a higher proliferation rate have a higher content than the slower-proliferating cells [36]. Selection for increased growth and breast muscle yield in turkey has resulted in conversion of the *p*. *major* muscle SCs to a population of cells with increased rates of proliferation and differentiation [19, 37, 38] and increased adipogenic potential [19, 22, 24, 39]. This difference can be further altered by environmental thermal stress [19, 22, 24, 37]. Changes in SC function and fate are likely, in part, the result of alterations in the SC populations that compose the *p*. *major* muscle.

Different populations of SCs can be identified by quantifying cell surface markers that are differentially expressed. One example in turkey SCs is a transmembrane heparan sulfate proteoglycan, syndecan-4 (SDC4) [40]. Syndecan-4 regulates proliferation [41–43] and migration [44] of turkey *p*. *major* muscle SCs, and it is highly expressed during proliferation but gradually decreases with differentiation [45]. In mammalian SCs, SDC4 regulates proliferation by promoting the progression of the cell cycle from G1- to S-phase [46]. The other major function of SDC4 is promoting SC migration by activating Ras homolog guanosine 5’-triphosphatase (Rho GTPase) family genes including Ras homolog gene member A (RhoA) [45] and Ras-related C3 botulinum toxin substrate 1 (Rac1) [47]. Migration is critical for the alignment of SCs prior the formation of multinucleated myotubes [48]. These studies suggest that SDC4 is an ideal cell surface marker representing both proliferation and migration ability of SCs.

In addition to SDC4, CD44 is also a potentially differentially expressed SC surface marker. As a transmembrane protein receptor, the extracellular domain of CD44 contains binding sites for hyaluronic acid (HA) [49] and osteopontin (OPN) [50]. Upon the binding of HA, the CD44 cytoplasmic domain mediates cell migration through Rac1 [51] and RhoA [52]. In skeletal muscle, CD44 has been reported to promote proliferation, migration, and myotube formation of myogenic cells through an interaction with HA [53, 54]. The interaction between HA and CD44 also has an important role in regulating the function of adipogenic cells [55]. As reported by Lee et al. [56], HA inhibits expression of adipogenic genes and decreases fat accumulation in mouse preadipocytes via a CD44-mediated mechanism. In addition, CD44 is responsible for insulin-resistance in adipose tissue, as CD44 deficiency reduces insulin-resistance while increasing lipid accumulation in mouse [55] and human [57] white fat tissue. In contrast, the interaction between CD44 and OPN has a positive effect on proliferation and adipogenesis of preadipocytes [58, 59]. Thus, CD44 can be regarded as a marker representing migration, myogenesis, and adipogenic potential of SCs.

We previously reported that both growth selection and thermal stress affects turkey SC proliferation, myogenesis, and adipogenesis especially during the period of peak SC mitotic activity and temperature sensitivity (the first week after hatch) [19, 22]. We also found changes in SC function and fate in response to growth selection and thermal stress are regulated by mechanistic target of rapamycin (mTOR) pathway [60, 61] and the Frizzled-7 (Fzd7)-mediated wingless-type mouse mammary tumor virus integration site family/planar cell polarity (Wnt/PCP) pathway [62]. Although SDC4 [41–44] and CD44 [53, 54] mediate SC activity, their differential roles in signal transduction pathways as well as in the impact of growth selection and thermal stress have not been studied. Modifications in SDC4 and CD44 expression may alter the function and fate of SCs and SC-mediated processes with a long-lasting impact on *p*. *major* muscle development, growth, morphology, and protein to fat ratio.

The objective of this study was to determine the differential effect of thermal stress on SDC4 and CD44 expression in growth-selected turkey *p*. *major* muscle SCs and how the protein expression is modulated by the mTOR and Wnt/PCP pathways during both proliferation and differentiation. To this end, SCs used in this study were isolated from the *p*. *major* muscle of 1-week modern meat-type commercial (NC) turkeys and 1-week historic Randombred Control Line 2 (RBC2) turkeys. The NC turkeys were selected for improved growth performance including increased growth rate and breast muscle yield, whereas the RBC2 turkeys are slower-growing turkeys representing commercial turkeys in 1960s [63]. Alteration of SDC4 and CD44 expression by mTOR and Wnt/PCP pathways is examined through knockdown of *mTOR* and *Fzd7* gene. Results of this study will show how SC populations differentially changed with thermal stress in growth-selected turkey *p*. *major* SCs, and how this phenomenon was related to changes in the expression of SDC4 and CD44.

## 2. Materials and methods

### 2.1. Satellite cells

Satellite cells were previously isolated from the *p*. *major* muscle of 1-week RBC2 and NC turkeys according to the method by Velleman et al. [38]. From each sample collected, all the SCs were passaged to a fourth pass and stored in liquid nitrogen until use. To avoid sex-related differences [38], only male turkeys were used for the cell isolation.

### 2.2. Satellite cell culture for thermal challenging

Satellite cells from both the RBC2 and NC turkey lines were plated on 6-well gelatin-coated plates (Corning, Corning, NY, USA) at 35, 000 cells per well in 4 mL of Dulbecco’s Modified Eagle’s Medium (DMEM, Thermo Fisher Scientific, Waltham, MA, USA) plating medium containing 10% chicken serum (Sigma-Aldrich, St. Louis, MO, USA), 5% horse serum (Sigma-Aldrich), 1% antibiotics-antimycotics (Corning), and 0.1% gentamicin (Gemini Bio-Products, West Sacramento, CA, USA). Plated cells were incubated at 38°C in a 95% air / 5% CO2 incubator (Thermo Fisher Scientific) for 24 h allowing for cell attachment. The plating medium was then replaced with McCoy’s 5A growth medium (Sigma-Aldrich) containing 10% chicken serum, 5% horse serum, 1% antibiotics-antimycotics, and 0.1% gentamicin.

For each experimental round, SC samples from both the RBC2 and NC line were randomly assigned to be incubated either at 38° and 43°C or at 38° and 33°C for 72 h of proliferation with the medium changed every 24 h. At 72 h of proliferation, the growth medium was replaced with a DMEM differentiation medium containing 3% horse serum, 1% antibiotics-antimycotics, 0.1% gentamicin, 0.1% gelatin, and 1 mg/mL bovine serum albumin (BSA, Sigma-Aldrich) and incubated at the same temperature as the temperature during proliferation for additional 48 h of differentiation. The differentiation medium was changed every 24 h. At both 72 h of proliferation and 48 h of differentiation (sampling times), plates were removed from the incubator, and rinsed twice with phosphate buffered saline (PBS, 171 mM NaCl, 3.35 mM KCl, 1.84 mM KH_2_PO_4_, and 10 mM Na_2_HPO_4_, pH7.08) for cell surface markers staining and flow cytometry. A total of 11 experimental rounds were conducted such that for each of the NC and RBC2 lines, all combinations of temperature and sampling times were run at least twice in random order.

### 2.3. Small interfering RNA and satellite cell transfection

Small interfering RNAs targeting *mTOR* (Gene bank ID: XM_010723001.3) and *Fzd7* (Gene bank ID: XM_010713460.1) were designed using Invitrogen Block-iT software (https://rnaidesigner.thermofisher.com/rnaiexpress/setOption.do?designOption=stealth&pid). The mTOR siRNA and Fzd7 siRNA were synthesized stealth duplex siRNAs (Thermo Fisher Scientific, MA, US). The mTOR siRNA targets the *mTOR* open reading frame from 3933 to 3957 with the following sequence: sense strand: 5’-CAA AGA UGA CUG GUU GGA AUG GUU A-3’; anti-sense strand: 5’-UUA CCA UUC CAA GUC AUC UUU G-3’. The Fzd7 siRNA targets the *Fzd7* open reading frame from 1522 to 1546 with the following sequence: sense strand: 5’-CCG GAC UUC ACA GUC UUC AUG AUC A-3’; anti-sense strand: 5’-UGA UCA UGA AGA CUG UGA AGU CCG G-3’. Knockdown efficiency of the synthesized mTOR siRNA [60] and Fzd7 siRNA [62] has been confirmed to be at least 50% in both the RBC2 and NC line SCs. A stealth siRNA with 48% guanine and cytosine content was used as the negative control siRNA (Thermo Fisher Scientific).

For each experimental round, SCs from each line were plated (38, 000 cells per well) in 6-well gelatin-coated plates in DMEM transfection medium (2 mL per well) supplemented with 10% chicken serum and 5% horse serum, and incubated at 38°C in a 95% air / 5% CO2 incubator for 24 h. After 24 h of attachment, cells in each well were transfected with 100 pmol of the mTOR siRNA, Fzd7 siRNA, or negative control siRNA with 5 μl of lipofectamine 2000 (Thermo Fisher Scientific) according to the manufacturer’s protocol. After 12 h of transfection, the plating medium was replaced with the growth medium. Satellite cells from both lines were then randomly assigned to proliferate either at 38° and 43°C or at 38° and 33°C for 72 h, with the growth medium changed every 24 h. After 72 h of proliferation, the growth medium was replaced with the differentiation medium for 48 h of differentiation. The differentiation medium was changed every 24 h. At 72 h of proliferation and 48 h of differentiation (sampling time), plates were removed from incubator, rinsed twice with PBS for staining of cell surface markers and flow cytometry. A total of 21 experimental rounds were conducted such that for each of the NC and RBC2 lines, all combinations of temperature, knockdown of gene, and sampling times were run at least twice in random order.

### 2.4. Staining of satellite cell surface markers and flow cytometry

For each experimental round, at either 72 h of proliferation or 48 h of differentiation, 400 μL of ice-cold 0.05% trypsin-EDTA (Thermo Fisher Scientific) was added to each culture well. After incubation for 3 min, SCs were scraped off from each plate with a cell scraper (Thermo Fisher Scientific) and transferred to a centrifuge tube containing 30 mL of ice-cold PBS to stop the enzyme reaction. Each centrifuge tube was then centrifuged at 135 g for 10 min. After centrifugation, supernatant was gently removed without affecting the pellet, and SC pellet was then resuspended in 400 μL of ice-cold PBS by pipetting. Cell suspension was transferred to a 1.5 mL microtube (USA Scientific, Ocala, FL, USA). Cell concentration was measured using a Coulter counter (Beckman Coulter Z2 Particle Counter and Size Analyzer, Brea, CA, USA) according to the manufacturer’s protocol, and was adjusted to 1,000,000 cells/mL with ice-cold PBS. In each treatment group, 3 μL of goat anti-SDC4 (Santa Cruz Biotechnology, Dallas, TX, USA) and 0.6 μL of PE-Cyanine5-conjugated mouse anti-CD44 (Thermo Fisher Scientific) primary antibody were both added to 300 μL of PBS-suspended SCs, and were incubated at 4°C for 2 h. After incubation, the SCs were centrifuged at 135 g for 10 min and the supernatant was removed without affecting the pellet. The SCs were then washed in 400 μL of ice-cold PBS by pipetting, centrifuged at 135 g for 10 min with the supernatant removed. After repeating the wash for three times, the SCs were resuspended in 500 μL of ice-cold PBS containing 0.5 μL of Alexa Fluor-488-conjugated donkey anti-goat secondary antibody (Abcam, Waltham, MA, USA) and incubated at 4°C for 1 h. After incubation, the SCs were centrifuged at 135 g for 10 min and the supernatant was removed without affecting the pellet. The SCs were then washed in 400 μL of ice-cold PBS, centrifuged at 135 g for 10 min with the supernatant removed. After being washed for three times, the cell pellet was resuspended in ice-cold PBS and was readjusted to a concentration of 500,000 cells/mL in each microtube for flow cytometry.

**Fig 1:**
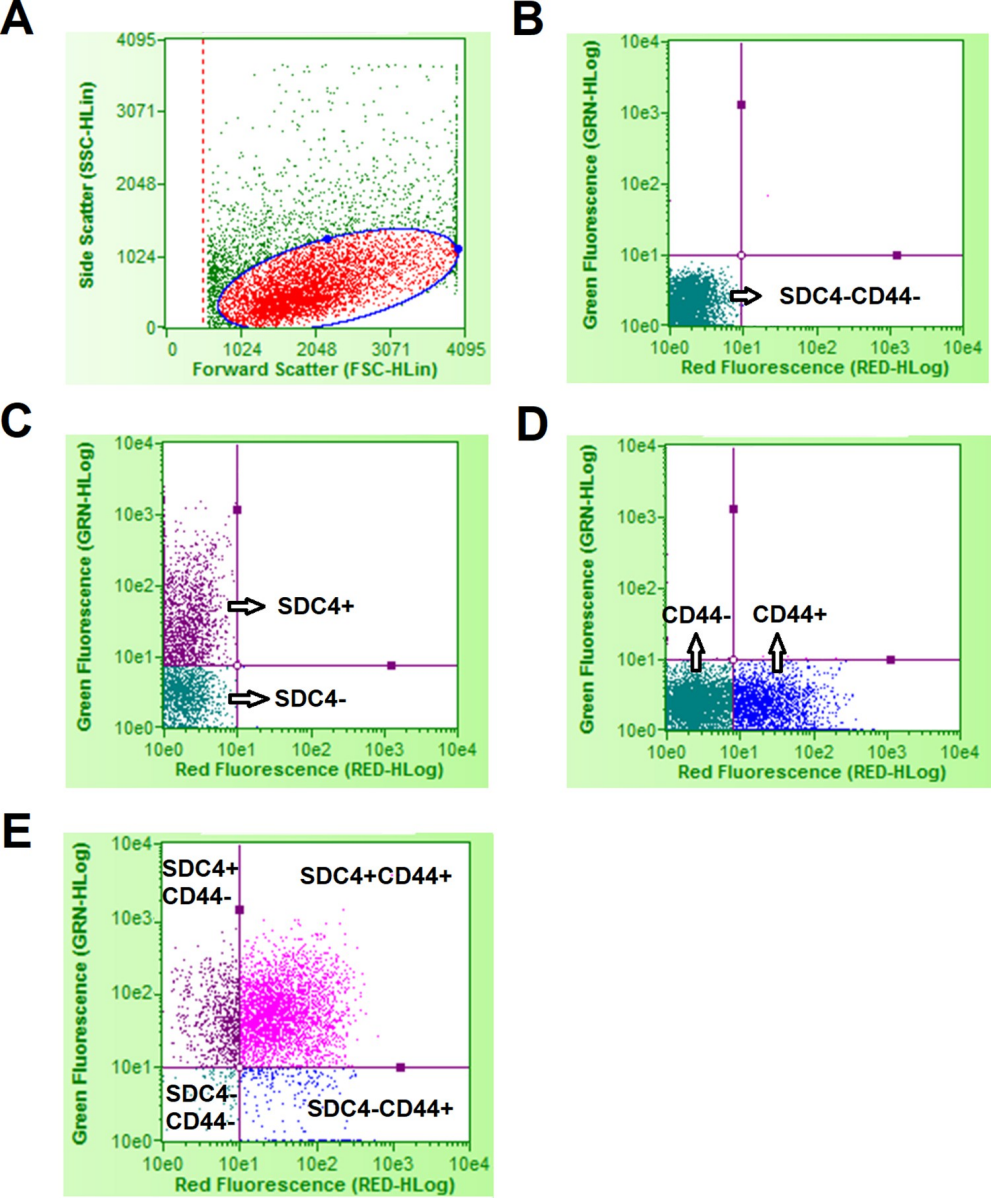
Flow cytometry gating strategy. Satellite cells were gated with an elliptical gate in a forward and side scatter plot to exclude cell debris (A). Within the gated cells, a quadrant was set to define the SDC4 and CD44 positive (+) and negative (−) satellite cells according to the fluorescent intensity of Alexa Fluor-488 (green fluorescence) and PE-Cyanine5 (red fluorescence). The position of the quadrant was determined by analyzing the satellite cells that were not incubated with any antibody (no fluorescence, B), incubated with the goat anti-SDC4 primary antibody followed by the Alexa Fluor-488-conjugated donkey anti-goat secondary antibody (green fluorescence only, C), or incubated with only the PE-Cyanine5-conjugated mouse anti-CD44 primary antibody (red fluorescence only, D). A representative sample of the SDC4 and CD44 double staining cells is shown in graph E.

The stained SCs in each microtube were analyzed with a Guava EasyCyte flow cytometer (MilliporeSigma, Burlington, MA, USA) according to manufacturer’s Guava ExpressPlus assay protocol. In brief, cells were gated with a forward and side scatter plot to exclude cell debris (Fig 1A). Within the gated cells, a quadrant was set to define the SDC4 and CD44 positive and negative cells according to the fluorescent intensity of Alexa Fluor-488 and PE-Cyanine5. The position of the quadrant was determined by analyzing the RBC2 line SCs that were not incubated with any antibody (no fluorescence, Fig 1B), incubated with the goat anti-SDC4 primary antibody followed by the Alexa Fluor-488-conjugated donkey anti-goat secondary antibody (green fluorescence only, Fig 1C), or incubated with only the PE-Cyanine5-conjugated mouse anti-CD44 primary antibody (red fluorescence only, Fig 1D). The quadrant-defined SC populations including SDC4 and CD44 double negative cells (SDC4^−^CD44^−^, lower left quadrant), SDC4 positive and CD44 negative cells (SDC4^+^CD44^−^, upper left quadrant), SDC4 negative and CD44 positive cells (SDC4^−^CD44^+^, lower right quadrant), and SDC4 and CD44 double positive cells (SDC4^+^CD44^+^, upper right quadrant). Each individual sample in a microtube was quantified for the relative proportion of a quadrant-defined SC population using the Guava EasyCyte system. Three readings were recorded on each sample (i.e. microtube). Quadrant-defined SC populations for one representative sample are shown in Fig 1E.

### 2.5. Statistical analysis

For each study, a general linear mixed model was fitted to each of the response variables, namely, the relative proportion of SDC4^−^CD44^−^, SDC4^+^CD44^−^, SDC4^−^CD44^+^, and SDC4^+^CD44^+^ SCs, assuming a normal distribution.

For the first study (described in section 2.2 and 2.4), the linear predictor included the fixed effects of sampling time (72 h of proliferation and 48 h of differentiation), temperature (33°, 38° and 43°C) and turkey cell line (RBC2 and NC), as well as all 2- and 3-way interactions. In addition, the linear predictor included the random effect of experimental round nested within sampling time and the random effect of microtube (identified by the cross product between experimental round, temperature and cell line) to reflect the experimental design. The latter random effect also recognized subsampling due to triplicate observations collected on each microtube.

For the second study (described in section 2.3 and 2.4), the linear predictor included the fixed effects of sampling time (72 h of proliferation and 48 h of differentiation), temperature (33°, 38° and 43°C), turkey cell line (RBC2 and NC), and knockdown of gene (Control, *Fzd7*, or *mTOR*), as well as all 2-way, 3-way, and 4-way interactions. Specification of random effects in the statistical model was as explained for the first study. Taken together, these model specifications enabled the recognition of the appropriate level of replication for each treatment factor for each study, thus allowing appropriate calibration of test statistics and estimation of degrees of freedom for inference. Model assumptions were evaluated using studentized residuals and were considered to be reasonably met.

All statistical models were fitted using the GLIMMIX procedure of SAS (Version 9.4, SAS Institute, Cary, NC). Variance components were estimated using restricted maximum likelihood. A Kenward-Rogers approach was used to estimate degrees of freedom and make corrections to estimated standard errors. When needed due to variance component estimates converging to zero, degrees of freedom were tuned manually. Estimated least square means and corresponding standard errors were presented at the appropriate level of inference in the factorial treatment structure. Relevant pairwise comparisons between treatment groups were conducted using a Bonferroni adjustment or a Dunnett adjustment (relative to the control) to avoid inflation of Type I error rate due to multiple comparisons. Adjusted *P*-values were used for inference, and *P* ≤ 0.05 was considered for reporting of significant differences between groups.

## 3. Results

### 3.1. Differential effects of thermal stress, and knockdown of *mTOR* or *Fzd7* on SDC4 and CD44 double negative SC population

#### 3.1.1. Line differences and temperature effects

Table 1 shows estimated proportion and standard error of SDC4^−^CD44^−^ SCs from RBC2 and NC cell lines incubated at selected temperatures for 72 h of proliferation followed by 48 h of differentiation. For the SDC4^−^CD44^−^ cells, interaction effects between temperature and cell line (*P* = 0.0268) and between temperature and sampling time (*P* < 0.0001) were apparent. That is, cell line differences in the proportion of SDC4^−^CD44^−^ cells were found to be specific to each temperature (33°, 38° or 43°C) whereas temperature effects were specific to each sampling time (72 h of proliferation or 48 h of differentiation).

**Table 1:** Estimated mean proportion (and corresponding standard error) of SDC4 and CD44 double-negative satellite cells in Randombred Control Line 2 (RBC2) and modern commercial line (NC) turkeys satellite cell populations incubated at selected incubation temperatures for 72 h proliferation and 48 h differentiation.

Sampling time	Temperature^1^	Cell line			
		RBC2		NC	
		EM^2^	SEM^3^	EM	SEM
72 h of Proliferation	33	0.94 a, x	0.16	0.75 b, x	0.16
	38	1.37 a, x	0.12	1.73 a, x	0.12
	43	1.17 a, x	0.16	1.20 ab, x	0.16
48h of Differentiation	33	1.51 a, x	0.16	0.93 ab, x	0.16
	38	1.53 a, x	0.13	1.43 a, x	0.13
	43	0.11 b, x	0.18	0.17 b, x	0.18
L × T^4^	*P =* 0.0268				
S × T^5^	*P* < 0.0001				

^1^ Incubation temperature (°C)

^2^ EM = estimated mean proportion of cells (%)

^3^ SEM = standard error of estimated mean

^4^ Interaction effect between cell line and temperature (L × T)

^5^ Interaction effect between sampling time and temperature (S × T)

a, b Different letters indicate differences between incubation temperatures within a cell line at a selected sampling time (vertical comparisons; *P* ≤ 0.05)

x, y Different letters indicate differences between cell lines within an incubation temperature at a selected sampling time (horizontal comparisons; *P* ≤ 0.05)

At 72 h of proliferation, no evidence for cell line differences in the proportion of SDC4^−^CD44^−^ cells was observed at neither of the temperatures considered 33°, 38° or 43°C (*P* ≥ 0.5012). For RBC2 SCs, there was no evidence for any temperature effects in the proportion of SDC4^−^CD44^−^ cells at 72 h of proliferation (*P* ≥ 0.2827). Yet, for NC SCs incubated for 72 h of proliferation, cold stress (33°C) decreased the proportion of SDC4^−^CD44^−^ cells compared to control (38°C) (*P* = 0.0005), while heat stress (43°C) showed no significant effect (*P* = 0.1990).

At 48 h of differentiation, line differences in the proportion of SDC4^−^CD44^−^ cells was not significant at 33°, 38° or 43°C (*P* ≥ 0.2307). In turn, the proportion of SDC4^−^CD44^−^ cells was decreased at 43°C compared to 38°C in both the RBC2 (*P* = 0.0002) and NC (*P* < 0.0001) cells, respectively. At 33°C, no significant change in the proportion of SDC4^−^CD44^−^ cells was observed in either line compared to controls at 38°C (*P* ≥ 0.3086).

#### 3.1.2. Effect of *mTOR* knockdown

Table 2 shows estimated proportion and standard error of SDC4^−^CD44^−^ SCs from RBC2 and NC cell lines challenged with hot or cold thermal stress, knocked down of *mTOR* or *Fzd7* gene, and incubated for 72 h of proliferation followed by 48 h of differentiation. For SDC4^−^CD44^−^ cells, 4-way interaction effects between sampling time, cell line, temperature, and knockdown of *mTOR* were significant (*P* < 0.0001, Table 2, S2 Fig). That is, the effect of *mTOR* knockdown on the proportion of SDC4^−^CD44^−^ SCs was specific to each cell line and differed between incubation temperatures and sampling times.

**Table 2:** Estimated mean proportion (and corresponding standard error) of SDC4 and CD44 double-negative satellite cells in satellite cell populations of Randombred Control Line 2 (RBC2) and modern commercial line (NC) turkeys subjected to knockdown of mTOR and Fzd7 at selected incubation temperatures for 72 h proliferation and 48 h differentiation.

Sampling time	Temperature^1^	Knockdown^2^	Cell line			
			RBC2		NC	
			EM^3^	SEM^4^	EM	SEM
72 h Proliferation	33	mTOR	0.17 #	0.19	0.12 #	0.19
		Control	1.25	0.15	0.98	0.15
		Fzd7	0.25 #	0.19	0.23 #	0.19
	38	mTOR	1.25 #	0.15	1.67 #	0.15
		Control	2.45	0.12	2.21	0.12
		Fzd7	0.19 #	0.16	0.21 #	0.16
	43	mTOR	2.02	0.19	1.23 *	0.19
		Control	1.83	0.17	1.28	0.17
		Fzd7	0.30 #	0.23	0.25 #	0.23
48h Differentiation	33	mTOR	0.10 #	0.19	0.15 #	0.19
		Control	1.21	0.16	0.79	0.16
		Fzd7	0.40 #	0.23	0.75	0.23
	38	mTOR	1.02	0.15	0.81	0.15
		Control	0.79	0.12	0.74	0.12
		Fzd7	2.04 #	0.17	1.36 #*	0.17
	43	mTOR	0.55 #	0.19	1.02 #	0.19
		Control	3.62	0.17	2.77 *	0.17
		Fzd7	0.52 #	0.23	0.55 #	0.23
S × L × T × K^5^			*P* < 0.0001			

^1^ Incubation temperature (°C)

^2^ Control = transfecting cells with a negative control small interfering RNA sequence; Fzd7 = knockdown of *Fzd7*; mTOR = knockdown of *mTOR*

^3^ EM = estimated mean proportion of cells (%)

^4^ SEM = standard error of estimated mean (SEM)

^5^ Interaction effect among sampling time, line, temperature, and knockdown (S × L × T × K)

# Indicates significant differences of knockdown groups relative to controls within a cell line and temperature combination at a selected sampling time (vertical comparisons; *P* ≤ 0.05)

* Indicates significant differences between cell lines for a knockdown group at a selected combination of incubation temperature and sampling time (horizontal comparisons; *P* ≤ 0.05)

At 72 h of proliferation, the proportion of SDC4^−^CD44^−^ cells in both the RBC2 and NC cell lines was decreased in *mTOR* knockdown groups compared to control groups at both 33°C (*P* ≤ 0.0002) and 38°C (*P* ≤ 0.0017) incubation temperatures. In contrast, at 43°C, knockdown of *mTOR* had no significant effect on the proportion of SDC4^−^CD44^−^ cells compared to the control groups in either cell line (*P* ≥ 0.6336, Table 2).

At 48 h of differentiation, the proportion of SDC4^−^CD44^−^ cells was significantly lower in the *mTOR* knockdown group compared to the control only in the RBC2 cells incubated at 33°C (*P* < 0.0001) and in both cell lines at 43°C (*P* < 0.0001, Table 2). Rather, at 38°C, the proportion of SDC4^−^CD44^−^ cells in the *mTOR* knockdown group was not significantly different from that of the control group in either line (*P* ≥ 0.2574, Table 2).

#### 3.1.3. Effect of *Fzd7* knockdown

For SDC4^−^CD44^−^ cells, 4-way interaction effects between sampling time, cell line, temperature, and knockdown of *Fzd7* were apparent (*P* < 0.0001, Table 2, S2 Fig). That is, the effect of *Fzd7* knockdown on the proportion of SDC4^−^CD44^−^ SCs was specific to each cell line evaluated at a given temperature and sampling time (Table 2).

At 72 h of proliferation, knockdown of *Fzd7* decreased the proportion of SDC4^−^CD44^−^ cells compared to the control group in both lines at all the temperatures (*P* ≤ 0.0002; Table 2), thought the reduction was greater in the RBC2 line compared to the NC line.

At 48 h of differentiation, knockdown of *Fzd7* decreased the proportion of SDC4^−^CD44^−^ cells only in the RBC2 line at 33°C (*P* = 0.0044) and in both lines at 43°C (*P* < 0.0001, Table 2). At 38°C, knockdown of *Fzd7* increased the proportion of SDC4^−^CD44^−^ cells compared to the control group in both lines (*P* ≤ 0.0023, Table 2).

### 3.2. Differential effects of thermal stress, and knockdown of *mTOR* or *Fzd7* on SDC4 negative and CD44 positive SC population

#### 3.2.1. Line differences and temperature effects

Table 3 shows estimated proportion and standard error of SDC4^−^CD44^+^ SCs for RBC2 and NC cell lines incubated at selected temperature treatments for 72 h of proliferation followed by 48 h of differentiation. For the SDC4^−^CD44^+^ SCs, an interaction effect among sampling time, temperature, and cell line was apparent SCs (*P* < 0.0001, Table 3, S1 Fig). In other words, cell line differences were specific to combinations of incubation temperature and sampling time; meanwhile temperature differences were also found to be specific to each cell line at a given sampling time.

**Table 3:** Estimated mean proportion (and corresponding standard error) of SDC4 negative and CD44 positive satellite cells in Randombred Control Line 2 (RBC2) and modern commercial line (NC) turkey satellite cell populations incubated at selected incubation temperatures for 72 h proliferation and 48 h differentiation.

Sampling time	Temperature^1^	Cell line			
		RBC2		NC	
		EM^2^	SEM^3^	EM	SEM
72 h of Proliferation	33	1.47 b, x	1.04	4.84 c, x	1.04
	38	5.25 b, x	0.74	10.26 b, y	0.74
	43	34.21 a, x	1.08	46.71 a, y	1.08
48h of Differentiation	33	14.20 c, x	1.04	29.20 b, y	1.04
	38	23.42 b, x	0.81	43.15 a, y	0.81
	43	39.83 a, x	1.33	42.31 a, x	1.33
S × L × T^4^	*P* < 0.0001				

^1^ Incubation temperature (°C)

^2^ EM = estimated mean proportion of cells (%)

^3^ SEM = standard error of estimated mean

^4^ Interaction effect among sampling time, line, and temperature (S × L × T)

a, b, c Different letters indicate differences between incubation temperatures within a cell line at a selected sampling time (vertical comparisons; *P* ≤ 0.05)

x, y Different letters indicate differences between cell lines within an incubation temperature at a selected sampling time (horizontal comparisons; *P* ≤ 0.05)

At 72 h of proliferation, no significant line difference was observed in the proportion of SDC4^−^CD44^+^ cells at 33°C (*P* = 0.6409). By contrast, at both 38° and 43°C, the NC line SCs were composed of a greater proportion of SDC4^−^CD44^+^ cells compared to the RBC2 line (*P* ≤ 0.0032). Heat stress of 43°C increased the proportion of the SDC4^−^CD44^+^ cells compared to the control temperature (38°C) in both the RBC2 and NC lines (*P* < 0.0001). In contrast, cold stress (33°C) decreased the proportion of SDC4^−^CD44^+^ cells compared to 38°C only in the NC line (*P* = 0.0119). A larger change in magnitude was observed at 43°C [estimated mean: 10.26 (38°C), 46.71 (43°C); 95% CI] compared to 33°C [estimated mean: 10.26 (38°C), 4.84 (33°C); 95% CI] in the NC line.

At 48 h of differentiation, the proportion of SDC4^−^CD44^+^ cells was greater in the NC line compared to the RBC2 line at both 33° and 38°C (*P* < 0.0001). No significant line difference was observed in the proportion of SDC4^−^CD44^+^ cells at 43°C (*P* = 0.9988). The proportion of SCD4^−^CD44^+^ cells in the RBC2 SCs was increased at 43°C compared to 38°C (*P* < 0.0001). For the NC line, heat stress showed no significant effect on the proportion of SDC4^−^CD44^+^ cells relative to control (*P* = 1.0000). During the cold stress (33°C), the proportion of SDC4^−^CD44^+^ SCs decreased in both lines compared to 38°C, respectively (*P* < 0.0001).

#### 3.2.2. Effect of *mTOR* knockdown

Table 4 shows estimated proportion and standard error of SDC4^−^CD44^+^ SCs for RBC2 and NC cell lines challenged with hot or cold thermal stress, knocked down of *mTOR* or *Fzd7* gene, and incubated for 72 h of proliferation followed by 48 h of differentiation. An interaction effect among sampling time, cell line, temperature, and knockdown of *mTOR* was significant on the proportion of SDC4^−^CD44^+^SCs (*P* < 0.0001, Table 4, S2 Fig). Thus, the effect of *mTOR* knockdown on the proportion of SDC4^−^CD44^+^ SCs was considered specific to combinations of sampling time, cell line, and incubation temperature.

**Table 4:** Estimated mean proportion (and corresponding standard error) of SDC4 negative and CD44 positive satellite cells in satellite cell populations of Randombred Control Line 2 (RBC2) and modern commercial line (NC) turkeys subjected to knockdown of mTOR and Fzd7 at selected incubation temperatures for 72 h proliferation and 48 h differentiation.

Sampling time	Temperature^1^	Knockdown^2^	Cell line			
			RBC2		NC	
			EM^3^	SEM^4^	EM	SEM
72 h Proliferation	33	mTOR	9.17 #	0.44	11.63 #*	0.48
		Control	1.77	0.34	5.79 *	0.35
		Fzd7	6.52 #	0.44	8.53 #*	0.44
	38	mTOR	5.26	0.32	35.91 #*	0.34
		Control	6.05	0.25	11.77 *	0.26
		Fzd7	3.14 #	0.34	2.89 #	0.35
	43	mTOR	13.32 #	0.44	29.46 #*	0.48
		Control	31.47	0.37	43.43 *	0.38
		Fzd7	3.02 #	0.53	7.50 #*	0.53
48 h Differentiation	33	mTOR	4.93 #	0.44	7.74 #*	0.48
		Control	15.72	0.37	25.93 *	0.38
		Fzd7	3.28 #	0.53	10.51 #*	0.53
	38	mTOR	3.91 #	0.32	5.34 #	0.34
		Control	21.33	0.27	32.49 *	0.28
		Fzd7	4.47 #	0.38	6.54 #*	0.38
	43	mTOR	2.52 #	0.44	7.99 #*	0.48
		Control	29.21	0.37	34.42 *	0.38
		Fzd7	6.37 #	0.53	10.13 #*	0.53
S × L × T × K^5^			*P* < 0.0001			

^1^ Incubation temperature (°C)

^2^ Control = transfecting cells with a negative control small interfering RNA sequence; Fzd7 = knockdown of *Fzd7*; mTOR = knockdown of *mTOR*

^3^ EM = estimated mean proportion of cells (%)

^4^ SEM = standard error of estimated mean (SEM)

^5^ Interaction effect among sampling time, line, temperature, and knockdown (S × L × T × K)

# Indicates significant differences of knockdown groups relative to controls within a cell line and temperature combination at a selected sampling time (vertical comparisons; *P* ≤ 0.05)

* Indicates significant differences between cell lines for a knockdown group at a selected combination of incubation temperature and sampling time (horizontal comparisons; *P* ≤ 0.05)

At 72 h of proliferation, knockdown of *mTOR* significantly increased the proportion of SDC4^−^CD44^+^ cells compared to control group in both lines at 33°C (*P* < 0.0001). At 38°C, the proportion of SDC4^−^CD44^+^ cells was increased in the mTOR knockdown group compared to the control group only in the NC line (*P* < 0.0001). At 43°C, knockdown of *mTOR* significantly reduced the proportion of SDC4^−^CD44^+^ cells compared to the control group in both lines (*P* < 0.0001).

At 48 h of differentiation, knockdown of *mTOR* decreased the proportion of SDC4^−^CD44^+^ cells in both lines compared to the control groups at all temperatures (*P* < 0.0001). The reduction was greater in the NC line [estimated mean: at 33°C: 25.93 (Control), 7.74 (mTOR); at 38°C: 32.49 (Control), 5.34 (mTOR); 95% CI] compared to the RBC2 line [estimated mean: at 33°C: 15.72 (Control), 4.93 (mTOR); at 38°C: 21.33 (Control), 3.91 (mTOR); 95% CI] at both 33° and 38°C.

#### 3.2.3. Effect of *Fzd7* knockdown

A significant interaction was observed among the effects of sampling time, cell line, temperature, and knockdown of *Fzd7* on the proportion of SDC4^−^CD44^+^ cells (*P* < 0.0001, Table 4, S2 Fig). Thus, effect of *Fzd7* knockdown on the proportion of SDC4^−^CD44^+^ SCs was considered specific to each sampling time, cell line, and temperature.

Specifically, at 72h of proliferation at 33C incubation temperature, the proportion of SDC4^−^CD44^+^ cells was higher in the *Fzd7* knockdown group compared to the control group for both lines (*P* < 0.0001). However, at higher incubation temperatures, knockdown of *Fzd7* expression decreased the proportion of SDC4^−^CD44^+^ cells in both cell lines compared to the control groups (i.e. 38°C: *P* < 0.0001; 43°C: *P* < 0.0001).

At 48 h of differentiation, the proportion of SDC4^−^CD44^+^ population was decreased in the *Fzd7* knockdown group compared to the control group in both lines regardless of incubation temperature (*P* < 0.0001). Yet, the *Fzd7-*knockdow-induced reduction was proportionally greater in the NC line [estimated mean: at 33°C: 25.93 (Control), 10.51 (Fzd7); at 38°C: 32.49 (Control), 6.54 (Fzd7); at 43°C: 43.42 (Control), 10.13 (Fzd7); 95% CI] compared to the RBC2 line [estimated mean: at 33°C: 15.72(Control), 3.28 (Fzd7); at 38°C: 21.33 (Control), 4.47 (Fzd7); at 43°C: 29.21 (Control), 6.37 (Fzd7); 95% CI] at all the temperatures.

### 3.3. Differential effects of thermal stress, and knockdown of *mTOR* or *Fzd7* on SDC4 positive and CD44 negative SC population

#### 3.3.1. Line differences and temperature effects

Table 5 shows estimated proportion and standard error of SDC4^+^CD44^−^ SCs for RBC2 and NC cell lines incubated at selected temperature treatments for 72 h of proliferation followed by 48 h of differentiation. A significant 3-way interaction was observed among sampling time, temperature, and cell line on the proportion of SDC4^+^CD44^−^ SCs (*P* < 0.0001, Table 5, S1 Fig). Thus, cell line differences were found to be specific to a given incubation temperature at a sampling time; meanwhile temperature differences were considered are specific to each cell line at a given sampling time.

**Table 5:** Estimated mean proportion (and corresponding standard error) of SDC4 positive and CD44 negative satellite cells in Randombred Control Line 2 (RBC2) and modern commercial line (NC) turkey satellite cell populations incubated at selected incubation temperatures for 72 h proliferation and 48 h differentiation.

Sampling time	Temperature^1^	Cell line			
		RBC2		NC	
		EM^2^	SEM^3^	EM	SEM
72 h of Proliferation	33	46.51 a, x	0.57	26.66 a, y	0.57
	38	28.47 b, x	0.39	22.88 b, y	0.39
	43	7.09 c, x	0.52	4.49 c, y	0.52
48h of Differentiation	33	12.63 a, x	0.56	10.82 a, x	0.56
	38	10.47 a, x	0.43	7.13 b, y	0.43
	43	2.23 b, x	0.64	2.30 c, x	0.64
S × L × T^4^	*P* < 0.0001				

^1^ Incubation temperature (°C)

^2^ EM = estimated mean proportion of cells (%)

^3^ SEM = standard error of estimated mean

^4^ Interaction effect among sampling time, line, and temperature (S × L × T)

a, b, c Different letters indicate differences between incubation temperatures within a cell line at a selected sampling time (vertical comparisons; *P* ≤ 0.05)

x, y Different letters indicate differences between cell lines within an incubation temperature at a selected sampling time (horizontal comparisons; *P* ≤ 0.05)

At 72 h of proliferation, the proportion of SDC4^+^CD44^−^ cells in the RBC2 line was greater than that in the NC line in all temperatures (*P* ≤ 0.0382). The magnitude of the observed difference between cell lines was most pronounced at incubation temperatures of 33°C (estimated mean: RBC2: 46.51, NC: 26.66; 95% CI), the cell line difference decreased as incubation temperatures increased to 38°C (estimated mean: RBC2: 28.47, NC: 22.88; 95% CI), and further to 43°C (estimated mean: RBC2: 7.09, NC: 4.49; 95% CI). For the temperature effects, proportion of SDC4^+^CD44^−^ cells decreased at 33°C (*P* ≤ 0.0001) and increased at 43°C (*P* < 0.0001) compared to 38°C in both lines.

At 48 h of differentiation, cell line difference in the proportion of SDC4^+^CD44^−^ cells was significant only at 38°C, and the NC line composed a lower proportion of SDC4^+^CD44^−^ cells than the RBC2 line (*P* = 0.0002). Heat stress (43°C) decreased the proportion of SDC4^+^CD44^−^ cells in both lines (*P* < 0.0001). Cold stress (33°C), in contrast, increased the proportion of SDC4^+^CD44^−^ cells only in the NC line (*P* = 0.0003).

#### 3.3.2. Effect of *mTOR* knockdown

Table 6 shows estimated proportion and standard error of SDC4^+^CD44^−^ SCs for RBC2 and NC cell lines challenged with hot or cold thermal stress, knocked down of *mTOR* or *Fzd7* gene, and incubated for 72 h of proliferation followed by 48 h of differentiation. The interaction among sampling time, cell line, temperature, and knockdown of *mTOR* was significant on the proportion of SDC4^+^CD44^−^ SCs (*P* < 0.0001, Table 6, S2 Fig). Thus, the effect of *mTOR* knockdown on the proportion of SDC4^+^CD44^−^ SCs was considered specific to each combination of cell line and temperature at a given sampling time.

**Table 6:** Estimated mean proportion (and corresponding standard error) of SDC4 positive and CD44 negative satellite cells in satellite cell populations of Randombred Control Line 2 (RBC2) and modern commercial line (NC) turkeys subjected to knockdown of mTOR and Fzd7 at selected incubation temperatures for 72 h proliferation and 48 h differentiation.

Sampling time	Temperature^1^	Knockdown^2^	Cell line			
			RBC2		NC	
			EM^3^	SEM^4^	EM	SEM
72 h Proliferation	33	mTOR	21.36 #	0.82	11.57 #*	0.82
		Control	50.56	0.59	27.88 *	0.59
		Fzd7	35.28 #	0.85	21.66 #*	0.85
	38	mTOR	33.98 #	0.58	8.85 #*	0.58
		Control	31.81	0.44	24.47 *	0.44
		Fzd7	60.29 #	0.66	62.52 #	0.66
	43	mTOR	24.16 #	0.82	9.87 #*	0.82
		Control	14.72	0.64	6.01 *	0.64
		Fzd7	55.13 #	1.04	32.48 #*	1.04
48h Differentiation	33	mTOR	25.04 #	0.82	26.91 #	0.82
		Control	13.55	0.64	12.96	0.64
		Fzd7	52.10 #	1.04	25.59 #*	1.04
	38	mTOR	54.25 #	0.58	44.78 #*	0.58
		Control	12.29	0.46	8.93 *	0.46
		Fzd7	62.25 #	0.74	40.67 #*	0.74
	43	mTOR	45.60 #	0.82	28.78 #*	0.82
		Control	20.74	0.64	13.41 *	0.64
		Fzd7	33.76 #	1.04	27.13 #*	1.04
S × L × T × K^5^			*P* < 0.0001			

^1^ Incubation temperature (°C)

^2^ Control = transfecting cells with a negative control small interfering RNA sequence; Fzd7 = knockdown of *Fzd7*; mTOR = knockdown of *mTOR*

^3^ EM = estimated mean proportion of cells (%)

^4^ SEM = standard error of estimated mean (SEM)

^5^ Interaction effect among sampling time, line, temperature, and knockdown (S × L × T × K)

# Indicates significant differences of knockdown groups relative to controls within a cell line and temperature combination at a selected sampling time (vertical comparisons; *P* ≤ 0.05)

* Indicates significant differences between cell lines for a knockdown group at a selected combination of incubation temperature and sampling time (horizontal comparisons; *P* ≤ 0.05)

At 72 h of proliferation, knockdown of *mTOR* decreased the proportion of SDC4^+^CD44^−^ cells compared to the control group at 33°C in both lines (*P* < 0.0001). At 38°C, the proportion of SDC4^+^CD44^−^ cells was increased in the RBC2 line (*P* = 0.0046) and reduced in the NC line (*P* < 0.0001) in the *mTOR* knockdown groups compared to control. At 43°C, knockdown of *mTOR* increased the proportion of SDC4^+^CD44^−^ cells in both lines (*P* ≤ 0.0004).

At 48 h of differentiation, the proportion of SDC4^+^CD44^−^ cells was higher in all the *mTOR* knockdown groups compared to the controls at all temperatures and in both lines (*P* < 0.0001). The increase was greater in the RBC2 line [estimated mean: at 38°C: 12.29 (Control), 54.25 (mTOR); at 43°C: 20.74 (Control), 45.60 (mTOR); 95% CI] compared to the NC line [estimated mean: at 38°C: 8.93 (Control), 44.78 (mTOR); at 43°C: 13.41 (Control), 28.78 (mTOR); 95% CI] at both 38° and 43°C.

#### 3.3.3. Effect of *Fzd7* knockdown

A significant interaction was observed among the effects of sampling time, cell line, temperature, and knockdown of *Fzd7* in the determination of the proportion of SDC4^+^CD44^−^ SCs (*P* < 0.0001, Table 6, S2 Fig). Therefore, effect of *Fzd7* knockdown on the proportion of SDC4^+^CD44^−^ SCs was specific to each combination of cell line and temperature at each sampling time.

At 72h of proliferation, knockdown of *Fzd7* decreased the proportion of SDC4^+^CD44^−^ cells in both lines compared to the control at 33°C (*P* < 0.0001) with a larger reduction seen in the RBC2 line. At both 38°C and 43°C, knockdown of *Fzd7* increased the proportion of SDC4^+^CD44^−^ cells in both lines (*P* < 0.0001).

At 48 h of differentiation, knockdown of *Fzd7* increased the proportion of SDC4^+^CD44^−^ cells compared to the control group in both cell lines at all the temperatures (*P* < 0.0001). The magnitude of the increase was greater in the RBC2 line [estimated mean: at 33°C: 13.55 (Control), 52.10 (Fzd7); at 38°C: 12.29 (Control), 62.25 (Fzd7); 95% CI] compared to the NC line [estimated mean: at 33°C: 12.96 (Control), 25.59 (Fzd7); at 38°C: 8.93 (Control), 40.67 (Fzd7); 95% CI] at both 33° and 38°C.

### 3.4 Differential effects of thermal stress, and knockdown of *mTOR* or *Fzd7* on SDC4 and CD44 double positive SC population

#### 3.4.1. Line differences and temperature effects

Table 7 shows estimated proportion and standard error of SDC4^+^CD44^+^ SCs for RBC2 and NC cell lines challenged with hot or cold thermal stress, knocked down of *mTOR* or *Fzd7* gene, and incubated for 72 h of proliferation followed by 48 h of differentiation. A significant 3-way interaction was observed among sampling time, temperature, and cell line on the proportion of SDC4^+^CD44^+^ SCs (*P* < 0.0001, Table 7, S1 Fig). Thus, cell line differences were found to be specific to combinations of incubation temperature and sampling time, meanwhile, temperature differences were specific to a cell line at a given sampling time.

**Table 7:** Estimated mean proportion (and corresponding standard error) of SDC4 and CD44 double-positive satellite cells in Randombred Control Line 2 (RBC2) and modern commercial line (NC) turkey satellite cell populations incubated at selected incubation temperatures for 72 h proliferation and 48 h differentiation.

Sampling time	Temperature^1^	Cell line			
		RBC2		NC	
		EM^2^	SEM^3^	EM	SEM
72 h of Proliferation	33	51.13 c, x	1.10	67.79 a, y	1.10
	38	64.89 a, x	0.77	65.14 a, x	0.77
	43	57.47 b, x	1.10	47.54 b, y	1.10
48h of Differentiation	33	71.74 a, x	1.10	59.11 a, y	1.10
	38	64.58 b, x	0.85	48.30 c, y	0.85
	43	57.73 c, x	1.34	55.06 b, x	1.34
S × L × T^4^	*P* < 0.0001				

^1^ Incubation temperature (°C)

^2^ EM = estimated mean proportion of cells (%)

^3^ SEM = standard error of estimated mean

^4^ Interaction effect among sampling time, line, and temperature (S × L × T)

a, b, c Different letters indicate differences between incubation temperatures within a cell line at a selected sampling time (vertical comparisons; *P* ≤ 0.05)

x, y Different letters indicate differences between cell lines within an incubation temperature at a selected sampling time (horizontal comparisons; *P* ≤ 0.05)

At 72 h of proliferation following incubation at 38C, there was no evidence for line difference in the estimated proportion of SDC4^+^CD44^+^ cells (*P* = 1.0000). However, at 43°C, the proportion of SDC4^+^CD44^+^ cells was greater in the RBC2 line compared to the NC line (*P* < 0.0001). In contrast, at 33°C, the NC line SCs contain a larger proportion of SDC4^+^CD44^+^ cells (*P* < 0.0001) than RBC2. The proportion of SDC4^+^CD44^+^ cells significantly decreased at both 43°C (*P* = 0.0004) and 33°C (*P* < 0.0001) compared to the control temperature (38°C) in the RBC2 line at 72 h of proliferation. Meanwhile, in the NC line SCs, heat stress decreased the proportion of SDC4^+^CD44^+^ cells (*P* < 0.0001), while cold stress showed no significant effect (*P* = 0.8418).

At 48 h of differentiation, the proportion of SDC4^+^CD44^+^ cells was lower in the NC line compared to the RBC2 line at both 38°C (*P* < 0.0001) and 33°C (*P* < 0.0001). However, no significant line difference was observed at 43°C (*P* = 0.9966). The magnitude of the observed difference between cell lines was more pronounced at 38°C (estimated mean: RBC2: 64.58, NC: 48.30; 95% CI) compared to that at 33°C (estimated mean: RBC2: 71.74, NC: 59.11; 95% CI). For the temperature effect, the proportion of SDC4^+^CD44^+^ cells was increased at 33°C (*P* ≤ 0.0009) compared to the 38°C in both cell lines. At 43°C, the proportion of SDC4^+^CD44^+^ cells was reduced in the RBC2 line (*P* = 0.0076) and was increased in the NC line (*P* = 0.0088), compared to the 38°C, respectively.

#### 3.4.2. Effect of *mTOR* knockdown

Table 8 shows estimated proportion and standard error of SDC4^+^CD44^+^ SCs for RBC2 and NC cell lines challenged with hot or cold thermal stress, knocked down of *mTOR* or *Fzd7* gene, and incubated for 72 h of proliferation followed by 48 h of differentiation. A significant interaction was observed among the effects of sampling time, cell line, temperature, and knockdown of *mTOR on* the proportion of SDC4^+^CD44^+^ SCs (*P* < 0.0001, Table 8, S2 Fig). Thus, the effect of *mTOR* knockdown on the proportion of SDC4^+^CD44^+^ SCs was considered specific to each combination of cell line and temperature at a given sampling time.

**Table 8:** Estimated mean proportion (and corresponding standard error) of SDC4 and CD44 double-positive satellite cells in satellite cell populations of Randombred Control Line 2 (RBC2) and modern commercial line (NC) turkeys subjected to knockdown of mTOR and Fzd7 at selected incubation temperatures for 72 h proliferation and 48 h differentiation.

Sampling time	Temperature^1^	Knockdown^2^	Cell line			
			RBC2		NC	
			EM^3^	SEM^4^	EM	SEM
72 h Proliferation	33	mTOR	69.48 #	0.79	76.87 #*	0.79
		Control	46.38	0.56	65.32 *	0.56
		Fzd7	57.70 #	0.79	69.32 #*	0.79
	38	mTOR	59.64	0.56	53.70 #*	0.56
		Control	59.69	0.41	61.55 *	0.41
		Fzd7	36.22 #	0.62	34.24 #	0.62
	43	mTOR	60.57 #	0.79	59.51 #	0.79
		Control	52.02	0.62	49.31 *	0.62
		Fzd7	41.55 #	0.97	59.78 #*	0.97
48 h Differentiation	33	mTOR	69.60	0.79	64.87 #*	0.79
		Control	69.41	0.62	60.21 *	0.62
		Fzd7	44.43 #	0.97	63.36 #*	0.97
	38	mTOR	40.72 #	0.56	48.97 #*	0.56
		Control	65.59	0.43	57.84 *	0.44
		Fzd7	31.39 #	0.69	51.57 #*	0.69
	43	mTOR	51.46 #	0.79	62.34 #*	0.79
		Control	46.55	0.62	49.53 *	0.62
		Fzd7	59.45 #	0.97	62.28 #	0.97
S × L × T × K^5^			*P* < 0.0001			

^1^ Incubation temperature (°C)

^2^ Control = transfecting cells with a negative control small interfering RNA sequence; Fzd7 = knockdown of *Fzd7*; mTOR = knockdown of *mTOR*

^3^ EM = estimated mean proportion of cells (%)

^4^ SEM = standard error of estimated mean (SEM)

^5^ Interaction effect among sampling time, line, temperature, and knockdown (S × L × T × K)

# Indicates significant differences of knockdown groups relative to controls within a cell line and temperature combination at a selected sampling time (vertical comparisons; *P* ≤ 0.05)

* Indicates significant differences between cell lines for a knockdown group at a selected combination of incubation temperature and sampling time (horizontal comparisons; *P* ≤ 0.05)

At 72 h of proliferation, knockdown of mTOR increased the proportion of SDC4^+^CD44^+^ cells in both lines at both 33° and 43°C. At 38°C, the proportion of SDC4^+^CD44^+^ cells was lower in in the *mTOR* knockdown group compared to the control group only in the NC line (*P* < 0.0001).

At 48 h of differentiation, knockdown of *mTOR* increased the SDC4^+^CD44^+^ cells compared to the control only in the NC line at 33°C (*P* < 0.0001). At 38°C, the proportion of SDC4^+^CD44^+^ cells was decreased in the *mTOR* knockdown group compared to the control group in both lines [estimated mean: RBC2: 65.59 (Control), 40.72 (mTOR); NC: 57.84 (Control), 48.97 (mTOR); 95% CI, *P* < 0.0001]. However, knockdown of *mTOR* increased the proportion of SDC4^+^CD44^+^ cells in both lines at 43°C [estimated mean: RBC2: 46.55 (Control), 51.46 (mTOR); NC: 49.53 (Control), 62.34 (mTOR); 95% CI, *P* < 0.0001].

#### 3.4.3. Effect of *Fzd7* knockdown

An interaction among sampling time, cell line, temperature, and knockdown of *Fzd7 had a* significant effect on the proportion of SDC4^+^CD44^+^ SCs (*P* < 0.0001). Therefore, the effect of *Fzd7* knockdown on the proportion of SDC4^+^CD44^+^ SCs was considered specific to the combination of cell line and temperature at each sampling time (Table 8, S2 Fig).

At 72 h of proliferation incubated at 33C, knockdown of *Fzd7* increased the proportion of SDC4^+^CD44^+^ cells compared to the control group in both lines (*P* ≤ 0.0001). However, at 38C incubation, the proportion of SDC4^+^CD44^+^ cells was reduced in the *Fzd7* knockdown group compared to the control group in both lines (*P* < 0.0001). At 43°C, the proportion of SDC4^+^CD44^+^ cells was decreased in the RBC2 line SCs in response to the *Fzd7* knockdown (*P* < 0.0001) whereas it was increased in the NC line when compared to the control group (*P* < 0.0001).

At 48 h of differentiation at 33 C incubation temperature, knockdown of *Fzd7* decreased the proportion of SDC4^+^CD44^+^ cells in the RBC2 line (*P* < 0.0001) and increased the proportion of SDC4^+^CD44^+^ cells in the NC line (*P* = 0.0144) compared to the control groups. At 38°C, the proportion of SDC4^+^CD44^+^ cells was reduced in the *Fzd7* knockdown group compared to the control group in both lines (*P* < 0.0001), though the magnitude of the reduction was greater in the RBC2 line compared to the NC line [estimated mean: RBC2: 65.59 (Control), 31.39 (Fzd7); NC: 57.84 (Control), 51.57 (Fzd7); 95% CI]. However, at 43°C, the proportion of SDC4^+^CD44^+^ cells was increased (*P* < 0.0001) in the *Fzd7* knockdown group compared to the control group in both lines [estimated mean: RBC2: 46.55 (Control), 59.45 (Fzd7); NC: 49.53 (Control), 62.28 (Fzd7); 95% CI].

## 4. Discussion

Throughout the past few decades, intensive growth selection of poultry has resulted in faster-growing lines with increased growth rate and breast muscle yield compared to slower-growing lines [9, 10]. Growth-selected turkeys also have increased myofiber diameter and reduced connective tissue spacing in the *p*. *major* muscle compared to non-selected turkeys [64]. Early post-hatch heat stress further increases myofiber diameter [7] and stimulates intramuscular fat deposition [4, 5] in the *p*. *major* muscle of rapid-growing poultry lines resulting in undesirable changes in meat quality. These changes in *p*. *major* muscle development, growth, and morphology may be related to changes in the SC populations comprising the *p*. *major* muscle. Indeed, our previous *in vitro* studies have shown that SCs from faster-growing NC turkeys have higher proliferation and differentiation rates, more lipid content, and their function and fate are more sensitive to thermal stress than those of the slower-growing RBC2 turkeys [19, 22].

Satellite cells are composed of a heterogeneous population of cells [26, 28, 29]. Satellite cells isolated from the same muscle can vary in their rates of proliferation and differentiation [33] and in growth factor responsiveness [34, 35]. Different populations of SCs also express different genes including myogenic regulatory genes [29], adipogenic regulatory genes [36], and the genes encoding cell surface markers [65]. Results of the current study showed that SDC4 was differentially expressed in the NC and RBC2 SC populations. A major function of SDC4 in turkey SCs is to promote cell migration [45]. Syndecan-4 cytoplasmic domain has been reported to stimulates SC migration by activating the GTPases RhoA [45] and Rac1 [47]. Migration is required for SC alignment before fusion to form multinucleated myotubes [48], and thus, is an essential cellular process for myogenesis. Since the NC line SCs differentiate earlier than the RBC2 line [19], the migration velocity of the NC line SCs may also be greater than that of the RBC2 line. Results from the current study, however, showed that the faster-proliferating NC line SCs comprised a lower proportion of SDC4^+^CD44^−^ cells than those of the slower-proliferating RBC2 line during both proliferation and differentiation. The proportion of the SDC4^+^CD44^−^ cells further reduced with heat stress in both cell lines. Similarly, results of transcriptome analysis by Reed et al. [66] showed that the expression of *SDC4* gene was significantly lower in the NC line SCs compared to the RBC2 line, and was further decreased with a heat stress during proliferation. These results suggest SDC4 may not be the only cell surface marker regulating the migration of turkey SCs.

In addition to SDC4, CD44 has also been reported to promote the proliferation, migration, and myotube formation of myogenic cells in mammalian skeletal muscle [53, 54]. Like SDC4, the CD44 cytoplasmic domain is reported to mediate cell migration through Rac1 [51] and RhoA [52] in human tumor cell lines. As reported by Mylona et al. [53], SCs isolated from CD44-knockout mice showed decreased migration velocity at an early stage of differentiation, and thus, delayed subsequent myotube formation. In turkey SCs, expression of *CD44* gene significantly increased with heat stress in proliferating RBC2 and NC line SCs, and was greater in the NC line compared to the RBC2 line particularly under heat stress [66]. In the current study, SCs from the faster-proliferating NC turkeys have a higher proportion of SDC4^−^CD44^+^ cells compared to the RBC2 line during both proliferation and differentiation. In response to elevated temperature, the proportion of SDC4^−^CD44^+^ cells further increased in both lines during proliferation. Although the proportion of the SDC4^+^CD44^+^ cell also showed cell line-specific variation in response to thermal stress, the magnitude of the change was not as large as for SDC4^−^CD44^+^ cells. It is possible that the increased expression of *CD44* gene and the elevated proportion of SDC4^−^CD44^+^ cells during heat stress increased proliferation and migration, thereby likely promoting hypertrophic growth of myofibers leading to the formation of larger diameter myofibers in the *p*. *major* muscle of fast-growing turkeys.

Expression of CD44 is also involved in regulating adipogenesis in conjunction with HA [56] and OPN [58, 59] in preadipocytes. As reported by Xu et al. [22], the NC line SCs synthesized more lipids than the RBC2 line, and heat stress further increased the lipid content in cells of both lines. Increased lipid content may, in part, result from increased adipogenesis mediated by an increased proportion of SDC4^−^CD44^+^ cells. In the *p*. *major* muscle, increased adipogenesis by SCs has been linked to increased intramuscular fat depots [4, 5]. Thus, thermal stress during the period of SC peak mitotic activity can be expected to promote intramuscular fat deposition, at least, in part, by increasing the proportion of SDC4^−^CD44^+^ cells particularly in faster-growing turkeys. Future studies will be needed to assess whether HA and OPN are involved in the adipogenesis of turkey SCs through the CD44 receptor and how these are potentially affected during thermal stress and growth selection.

The present study further demonstrated the significance of the mTOR pathway in regulating the expression of SDC4 and CD44. Specifically, knockdown of *mTOR* differentially changed the expression of SDC4 and CD44 in turkey SCs with line differences specific to cellular developmental stage (i.e. proliferation vs. differentiation). A major function of the mTOR pathway in skeletal muscle is promoting myofiber hypertrophy and muscle mass accretion through protein synthesis [67, 68]. The mTOR pathway is also involved in regulating myogenesis [60, 69] and adipogenesis [61, 70] of SCs. As reported by Xu et al. [60], the activity of mTOR, namely phosphorylation, is generally increased in the NC line SCs compared to RBC2 cells. In addition, SDC4 has been shown to activate the mTOR pathway through phosphatidylinositol 3 kinase/protein kinase B (PI3K/Akt) signaling [71, 72]. In the current study, knockdown of *mTOR* increased the proportion of SDC4^+^CD44^−^ cells in both lines at all temperatures during differentiation, with a greater increase observed in the RBC2 line. The increased proportion of SDC4^+^CD44^−^ cells may be associated with an increased expression of SDC4. However, the increase in SDC4^+^CD44^−^ cells was not enough to compensate for the effect of *mTOR* knockdown on the function and fate of turkey SCs because knockdown of *mTOR* suppresses proliferation, differentiation, and adipogenesis in turkey SCs [60, 61]. Notably, the proportion of SDC4^−^CD44^+^ cells decreased with knockdown of *mTOR* in both lines at all incubation temperatures during differentiation, and the response of the NC line SCs was more sensitive to the knockdown of *mTOR* compared to the RBC2 line at both the control and lower temperatures. Since CD44 regulates the proliferation and migration of SCs [53, 54], decreased SC activity in response to the *mTOR* knockdown can be expected to associate with a decreased proportion of SDC4^−^CD44^+^ cells. Taken together, these results suggest that SDC4 and CD44 differentially expresses in RBC2 and NC line turkey *p*. *major* muscle SCs, and this seems to be, at least in part, regulated by the mTOR pathway. Changes in SDC4 and CD44 expression in the *p*. *major* muscle SCs during growth selection may result in the alteration of myofiber diameter, muscle mass accretion, and protein to fat ratio in the *p*. *major* muscle in an mTOR-dependent manner.

The Fzd7-mediated Wnt/PCP pathway is also important in regulating proliferation, differentiation, and adipogenesis of turkey *p*. *major* muscle SCs during both growth selection and thermal stress [62]. In this study, knockdown of *Fzd7* differentially altered expression of both SDC4 and CD44 in the RBC2 and NC line SCs. Specifically, in response to *Fzd7* knockdown, the proportion of SDC4^+^CD44^−^ cells increased at all the incubation temperatures during differentiation, with a greater increase observed in the RBC2 line. In mouse SCs, SDC4 is a co-receptor presenting a protein ligand Wnt7a to the Fzd7 receptor to initiate the Wnt/PCP pathway [73]. Knockdown of *Fzd7* may trigger the expression of SDC4 and increase the proportion of SDC4^+^CD44^−^ cells. However, the increased expression of the SDC4 could not effectively compensate for the decreased level of Fzd7, because decreased proliferation, differentiation, and adipogenesis has been observed in the *Fzd7* knockdown groups in both the RBC2 and NC line SCs [62]. In contrast, knockdown of *Fzd7* decreased the proportion of SDC4^−^CD44^+^ cells at all the temperatures, and a greater reduction was observed in the NC line SCs. Thus, the Fzd7-mediated Wnt/PCP pathway may regulate expression of both SDC4 and CD44 in turkey *p*. *major* muscle SCs in a growth-dependent manner. With a greater Wnt/PCP pathway activity and a higher proportion of SDC4^−^CD44^+^ cells, the NC line SC population can be expected to be more likely to promote the hypertrophic growth of myofibers and stimulate intramuscular fat deposition.

In summary, selection for increased growth and breast muscle yield has changed the population of SCs comprising the turkey *p*. *major* muscle as reflected in differences in SDC4 and CD44 expression, temperature sensitivity, and response to the mTOR and Wnt/PCP pathway. Specifically, the NC line SCs contain a lower proportion of SDC4^+^CD44^−^ cells and a greater proportion of SDC4^−^CD44^+^ cells compared to the RBC2 line. However, line difference in the proportion of SDC4^−^CD44^−^ and SDC4^+^CD44^+^ cells was not as large as that of the SDC4^−^CD44^+^ and SDC4^+^CD44^−^ cells. Further, heat stress decreased the proportion of SDC4^+^CD44^−^ cells in both lines while cold stress had a stimulatory effect. In contrast, the proportion of SDC4^−^CD44^+^ cells increased with heat stress and decreased with cold stress in both lines. The NC line SCs, in general, were more sensitive to both hot and cold thermal stress in the expression of SDC4 and CD44 compared to the RBC2 line. Expression knockdown of either *mTOR* or *Fzd7* increased the proportion of SDC4^+^CD44^−^ cells and decreased the proportion of SDC4^−^CD44^+^ cells in both lines during differentiation, and line differences was specific to incubation temperatures. Changes in the population of SCs in the expression of SDC4 and CD44 may alter the function, fate, and temperature sensitivity of turkey SCs through the mTOR and Wnt/PCP pathways.

## Supporting information

S1 Fig Representative flow cytometry plots of syndecan-4 (SDC4) and CD44 stained satellite cells with thermal stress and growth selection. Randombred Control Line 2 (RBC2) and modern commercial (NC) line satellite cells were incubated at 38, 43, or 33°C for 72 h of proliferation followed by 48 h of differentiation. Within each plot, a relative proportion of SDC4 and CD44 double negative cells (SDC4^−^CD44^−^, lower left quadrant), SDC4 positive and CD44 negative cells (SDC4^+^CD44^−^, upper left quadrant), SDC4 negative and CD44 positive cells (SDC4^−^CD44^+^, lower right quadrant), and SDC4 and CD44 double positive cells (SDC4^+^CD44^+^, upper right quadrant) was recorded for statistical analysis.
(TIF)

S2 Fig Representative flow cytometry plots of syndecan-4 (SDC4) and CD44 stained satellite cells with thermal stress, growth selection, and knockdown of *mechanistic target of rapamycin* (*mTOR*) or *frizzled-7* (*Fzd7*). Randombred Control Line 2 (RBC2) and modern commercial (NC) line satellite cells were transfected with control small interfering RNA (control siRNA), mTOR siRNA, or Fzd7 siRNA at the beginning of proliferation, and then, incubated at 38, 43, or 33°C for 72 h of proliferation followed by 48 h of differentiation. Within each plot, a relative proportion of SDC4 and CD44 double negative cells (SDC4^−^CD44^−^, lower left quadrant), SDC4 positive and CD44 negative cells (SDC4^+^CD44^−^, upper left quadrant), SDC4 negative and CD44 positive cells (SDC4^−^CD44^+^, lower right quadrant), and SDC4 and CD44 double positive cells (SDC4^+^CD44^+^, upper right quadrant) was recorded for statistical analysis.
(TIF)
