# Supplementary figures and images for: Differential effects of temperature and mTOR and Wnt-planar cell polarity pathways on syndecan-4 and CD44 expression in growth-selected turkey satellite cell populations
Source: PLoS One. 2023 Feb 3;18(2):e0281350. doi: 10.1371/journal.pone.0281350 (PMC9897570; doi:10.1371/journal.pone.0281350)

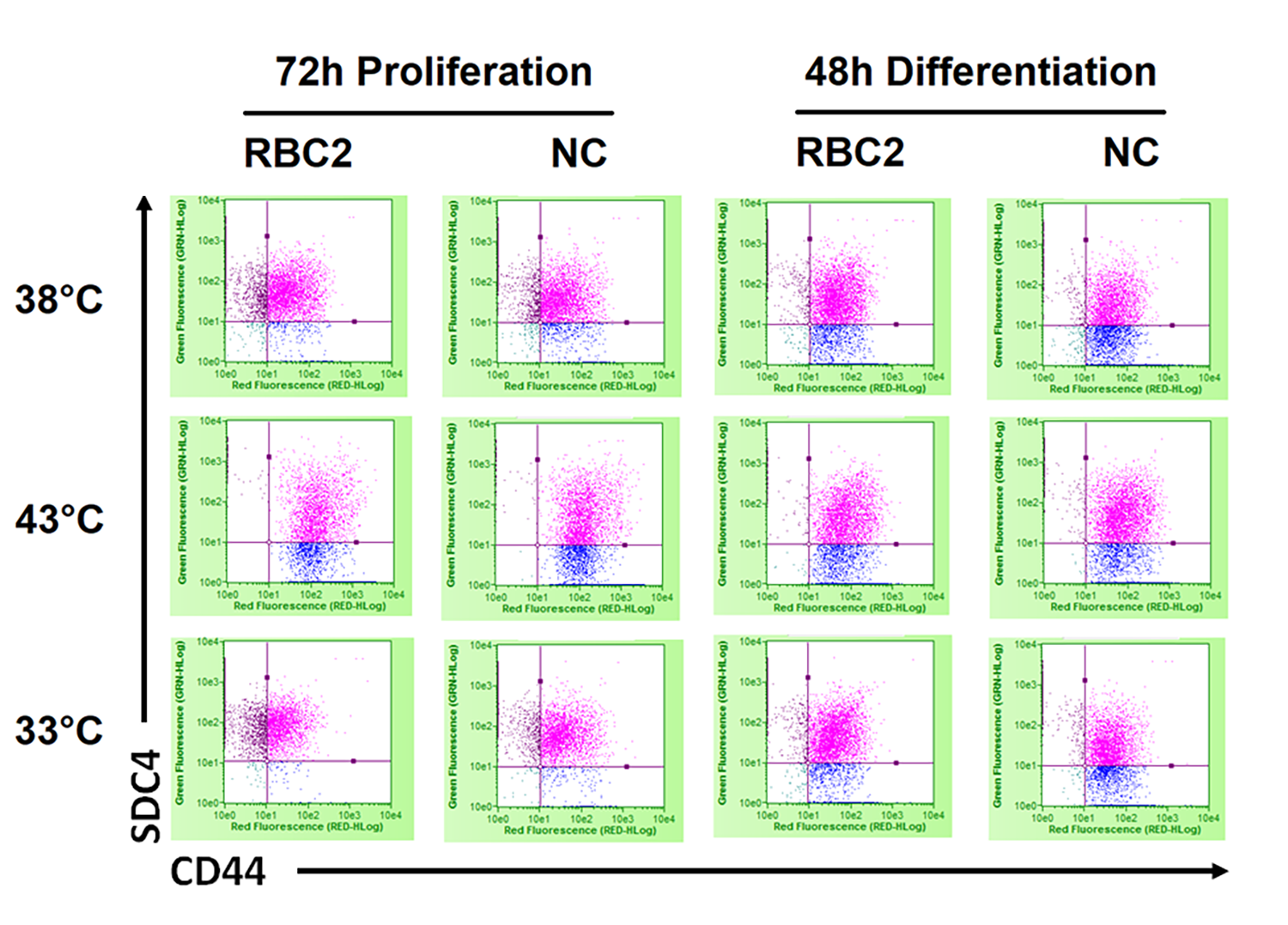

Supplement: S1 Fig — Randombred Control Line 2 (RBC2) and modern commercial (NC) line satellite cells were incubated at 38, 43, or 33°C for 72 h of proliferation followed by 48 h of differentiation. Within each plot, a relative proportion of SDC4 and CD44 double negative cells (SDC4−CD44−, lower left quadrant), SDC4 positive and CD44 negative cells (SDC4+CD44−, upper left quadrant), SDC4 negative and CD44 positive cells (SDC4−CD44+, lower right quadrant), and SDC4 and CD44 double positive cells (SDC4+CD44+, upper right quadrant) was recorded for statistical analysis. (TIF) [file pone.0281350.s001.tif]

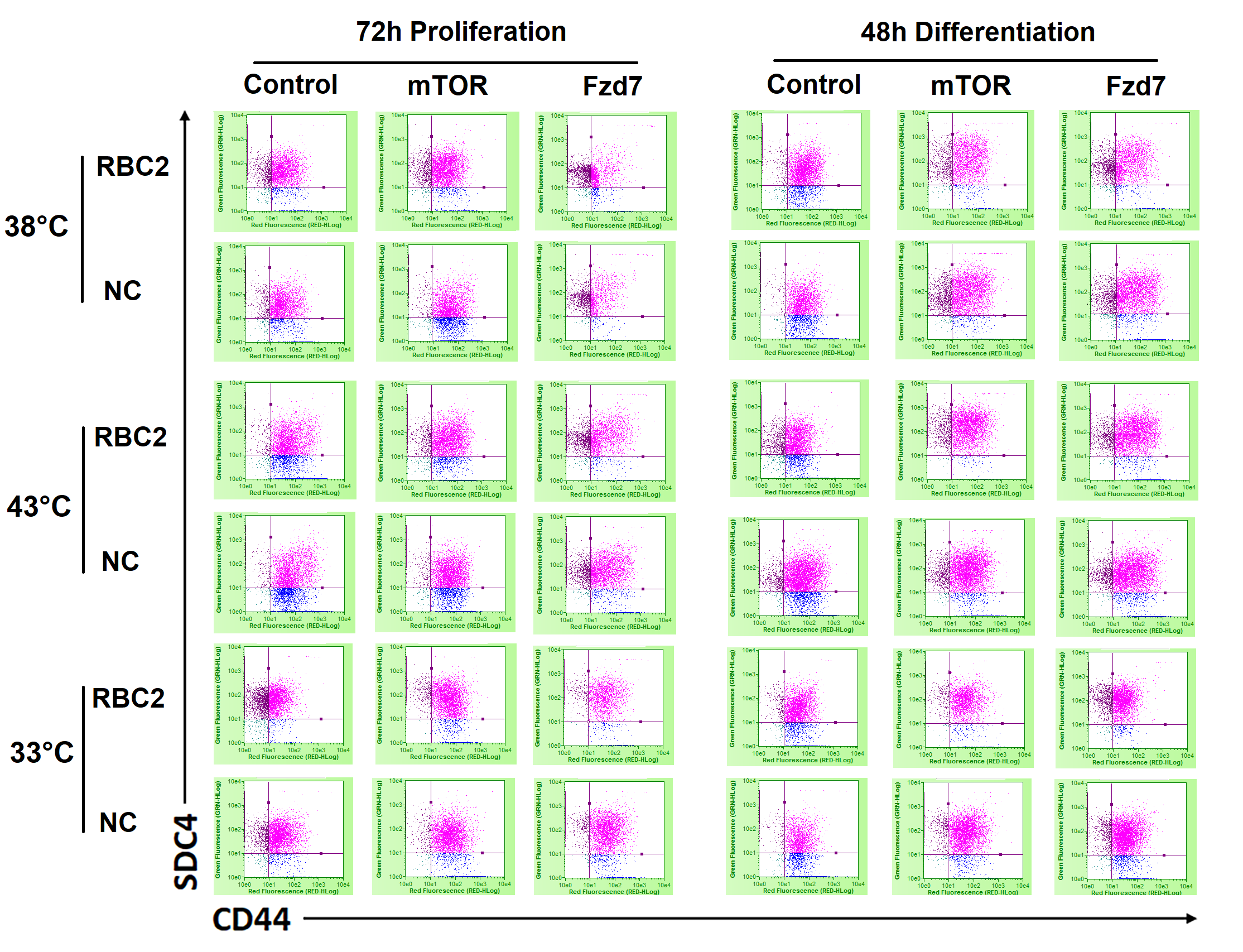

Supplement: S2 Fig — Randombred Control Line 2 (RBC2) and modern commercial (NC) line satellite cells were transfected with control small interfering RNA (control siRNA), mTOR siRNA, or Fzd7 siRNA at the beginning of proliferation, and then, incubated at 38, 43, or 33°C for 72 h of proliferation followed by 48 h of differentiation. Within each plot, a relative proportion of SDC4 and CD44 double negative cells (SDC4−CD44−, lower left quadrant), SDC4 positive and CD44 negative cells (SDC4+CD44−, upper left quadrant), SDC4 negative and CD44 positive cells (SDC4−CD44+, lower right quadrant), and SDC4 and CD44 double positive cells (SDC4+CD44+, upper right quadrant) was recorded for statistical analysis. (TIF) [file pone.0281350.s002.tif]
